# Integrons as surrogate marker for multi drug resistance in gram negative bacilli

**DOI:** 10.1186/1471-2334-12-S1-P72

**Published:** 2012-05-04

**Authors:** Gopalakrishnan Kaushik, K Arunkumar, G Sivakumar, R Rajasomasundharam, Padma Krishnan

**Affiliations:** 1 Dept of Microbiology, Dr ALM PG Institute of Basic Medical Sciences, University of Madras, Chennai, India; 2 Department of General Surgery, Madras Medical College, Chennai, India

## Background

Multi drug resistance (MDR) in gram negative bacilli (GNB) is an increasing global problem, which is often due to the acquisition of resistance genes from a shared pool. In MDR isolates, **integrons**-resistant gene cassettes, together with its accessory genetic elements (integrase) may be found in complex conglomerations on plasmids or on the chromosome and are widely acknowledged for their role in conferring MDR phenotype. Hence, the present study was done to evaluate the usefulness of integrase gene PCR as a surrogate marker for MDR in GNB.

## Methods

A total of 59 gram negative isolates collected between May 2010 and January 2011 from a tertiary hospital were included for this study. MDR phenotype of the isolates was established by the Kirby Bauer disc diffusion susceptibility test. Integrase gene PCR for class I and II integrons was performed.

## Results

Of the 59 isolates tested, *Pseudomonas* and *E. coli* were the predominant and 32 were MDR GNB. A total of 39 isolates were positive for either class I or class II integrons. Out of the 13 antibiotics tested, isolates carrying either class I or II integrons were resistant to 6 or more antibiotics, whereas isolates that were negative for carriage of integrons were resistant to 2 antibiotics or below.

## Conclusion

The study clearly indicates that integrons are widely prevalent in MDR GNB and further substantiates the usefulness of integrase gene PCR as a surrogate marker for MDR in GNB.

